# Rubber Trees Demonstrate a Clear Retranslocation Under Seasonal Drought and Cold Stresses

**DOI:** 10.3389/fpls.2016.01907

**Published:** 2016-12-20

**Authors:** Yuwu Li, Guoyu Lan, Yujie Xia

**Affiliations:** ^1^Key Laboratory of Tropical Forest Ecology, Xishuangbanna Tropical Botanical Garden, Chinese Academy of SciencesYunnan, China; ^2^Danzhou Investigation & Experiment Station of Tropical Crops, Ministry of Agriculture, Rubber Research Institute, Chinese Academy of Tropical Agricultural SciencesHainan, China; ^3^Kunming Institute of Zoology, Chinese Academy of SciencesYunnan, China

**Keywords:** rubber tree, nutrient strategy, leaf nutrient retranslocation, soil role, seasonal drought and cold stresses, soil microbial response

## Abstract

Having been introduced to the northern edge of Asian tropics, the rubber tree (*Hevea brasiliensis*) has become deciduous in this climate with seasonal drought and cold stresses. To determine its internal nutrient strategy during leaf senescence and deciduous periods, we investigated mature leaf and senescent leaf nutrients, water-soluble soil nutrients and characteristics of soil microbiota in nine different ages of monoculture rubber plantations. Rubber trees demonstrate complicated retranslocation of N, P, and K during foliar turnover. Approximately 50.26% of leaf nutrients and 21.47% of soil nutrients were redistributed to the rubber tree body during the leaf senescence and withering stages. However, no significant changes in the structure- or function-related properties of soil microbes were detected. These nutrient retranslocation strategy may be important stress responses. In the nutrient retranslocation process, soil plays a dual role as nutrient supplier and nutrient “bank.” Soil received the nutrients from abscised leaves, and also supplied nutrients to trees in the non-growth stage. Nutrient absorption and accumulation began before the leaves started to wither and fall.

## Introduction

Land-use changes increasingly threaten tropical forests, where biodiversity and human pressures are both high (Dirzo and Raven, 2003; Gibson et al., 2011; Li et al., 2013). Rapid conversion of tropical forests to agriculture, timber production or other uses has generated vast, human-dominated landscapes with negative consequences for tropical forest ecosystems (Gardner et al., 2009; Gibson et al., 2011). Rubber tree (*Hevea brasiliensis*) is an evergreen tree species in the rainforests of Amazon (Carr, 2012; Chen and Cao, 2015) and introduced to tropical Asia at the end of 19th century (Limkaisang et al., 2005). Global rubber plantations reached 10.06 million ha in 2010 (FAO, 2010) with 81% in South and Southeastern Asia (FAO, 2010). Indonesia, Malaysia, Thailand, and other countries are traditional planting regions, with expansion on the northern edge of Southeastern Asian tropics, especially in southwestern China, Laos, Cambodia, Myanmar, northeastern Thailand, and northwestern Vietnam. Presently, rubber plantations in these regions cover more than 1.5 million ha (Li and Fox, 2012).

The northern edge of Southeastern Asian tropics has a strong monsoon climate, dominated by warm-wet air masses from the Indian Ocean in summer and continental air masses from temperate regions in winter. About 80% of annual rainfall occurs during the rainy season from May to October; dry season (November to April) has only 20% of annual rainfall (Cao and Zhang, 1997; Liu et al., 2005; Cao et al., 2006). Formerly an evergreen species growing under moderate annual drought, rubber has adapted to withstand months of drought and cold stresses by becoming deciduous; annually shedding leaves in mid-dry season (late January and February). Previous studies on rubber plantations focused on impacts of land-use conversion on tropical biodiversity, soil and water conservation, and local climate change (Feng, 2007; Li et al., 2007; Powers et al., 2011; De Blécourt et al., 2013; Li, 2013; Xu et al., 2014; Li et al., 2015; Liu et al., 2016; Wu et al., 2016; Zhou et al., 2016). However, little research has been conducted on nutrient strategies of rubber trees and soil roles in these processes under drought and cold stresses; nor impacts on soil nutrients and microbiota.

Nutrient cycling is a vital function in both natural forests and man-made plantations. As sessile organisms, forest trees cope with persistently fluctuating availability of soil nutrients, both in space and time (Maillard et al., 2015). Nutrient strategies strongly affect plant performance. Facing nutrient deficiencies, different plant strategies have evolved to optimize acquisition and use of most macro- and some micronutrients. Litter fall is the major flux transferring nutrients to forest floor and soil (Parzych et al., 2008; Salehi et al., 2013). Most soil nutrients taken up by trees are used to annual produce foliage, which serves as a reservoir of reusable nutrients (Maillard et al., 2015). However, nutrients in one generation of foliage become sources with some retranslocated to support production of next-generation foliage (Malagoli et al., 2005; Abdallah et al., 2010). Before abscission, trees decrease leaf nutrient concentrations, especially before cold, drought, or other environmental stresses occur (Charley and Richards, 1983; Nambiar and Fife, 1987, 1991; Fife et al., 2008; Millard and Grelet, 2010). Retranslocation is an internal conservation process by which nutrients move from mature leaves into storage organs or growing tissues (Zhang et al., 2015). This can make a substantial contribution to annual nutrient use (Ryan and Bormann, 1982), can provide further nutrients for free uptake to ensure stability through cold or drought stress (Jha, 2014), and can make nutrients available for younger plant organs and contribute to nutrient-use efficiency (Himelblau and Amasino, 2001; Fischer et al., 2007; Avice and Etienne, 2014).

Facing up to 5 or 6 months (including cold foggy and dry periods from November to March or April of following year) of drought and cold stresses (Supplementary Figures S1 and S2), rubber trees are deciduous in the northern edge of the Southeastern Asian tropics. Here, rubber leaves are mature prior to mid-January, followed by total leaf losses, and in March, there is new leaf budding (Chen and Cao, 2008, 2015). Here also, rubber undergoes up to 8 months of annual latex harvest, with concomitant nutrient losses. We do not know when nutrient retranslocation by rubber trees begins. However, that nutrient loss ceases after latex harvesting, and nutrient strategies may differ from other evergreen and deciduous plant species, and possibly take soil as it’s own free-withdrawing nutrient “bank” in this period. Thus the following questions arise: what is soil’s role in the process of the rubber tree leaf nutrient retranslocation? Does rubber transfer nutrients from mature leaves to soil during leaf senescence and leaf-free periods as adaptations to these environmental pressures? How much do leaf processes account for total nutrient retranslocation efficiency (NRE)? Does this nutrient retranslocation strategy have impacts on soil microbes?

Tree age is another important factor affecting nutrient accumulation, distribution, and quantities of nutrients returned as forest litter (Ovington, 1968; Singh and Singh, 1991; Lau and Wong, 1993). Mature trees rely more on remobilization of nutrient stores for growth each spring than do juvenile trees (Millard et al., 2006; Maillard et al., 2015). Questions arise whether there are differences in nutrient retranslocation strategies or different impacts on soil nutrient bioavailability along rubber-plantation chronosequences.

**FIGURE 1:**
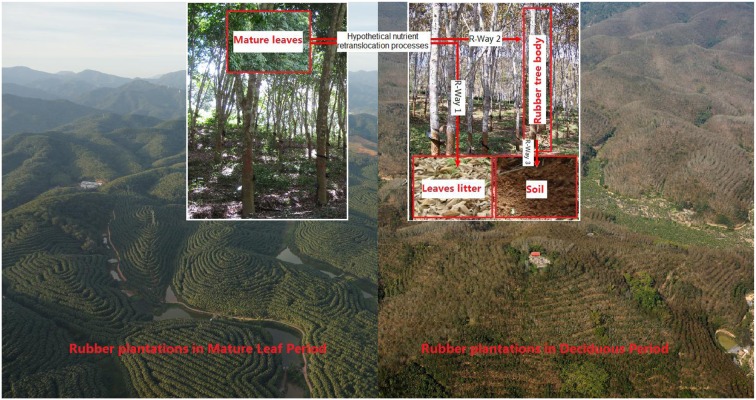
**Schematic diagram of the hypothetical nutrient retranslocation processes of rubber trees from mature leaf period to deciduous leaf period**.

In this study, we investigated mature leaf and senescent leaf (leaf litter) nutrients, water-soluble soil nutrients and some aspects of soil microbes in monoculture rubber plantations of nine different ages (1–48 years) in Xishuangbanna. This is a large rubber-plantation area on the northern tropical edge of Southeastern Asia. We hypothesized that rubber may use soil as a free-withdrawing nutrient “bank” during the post-harvest period. Rubber trees transfer nutrients from mature leaves to the tree body and soil during leaf senescence and deciduous periods as adaptations to these environmental pressures (**Figure 1**).

## Materials and Methods

### Study Site

The study was conducted in the Xishuangbanna National Nature Reserve (21°09′–22°33′N, 99°58′–101°34′E) in Southwest China, located at the northern edge of the Southeastern Asian tropics (Supplementary Figure S3). Located in the East Asian monsoon region, Xishuangbanna is dominated by warm, wet air masses from the Indian Ocean in summer and continental air masses from temperate regions in winter, resulting in a strongly seasonal climate. Dry season occurs from January to April, while the wet season is from May to October, and the foggy season is from November to December (Supplementary Figure S1; Cao and Zhang, 1997; Liu et al., 2005; Cao et al., 2006). The annual mean air temperature (at 2.2 m above surface) of rubber plantations is 21.1°C, and annual mean air temperature in rubber-tree canopy (at 25.1 m) is 22.0°C (2010–2015). The mean annual rainfall is 1,307 mm (2005–2015; Supplementary Figure S2). Rainfall during the foggy and dry seasons is only 20.06% of total annual rainfall, and the minimum monthly rainfall occurs in February (averaging 17.7 mm; Supplementary Figure S2). Patterns of precipitation and soil water content were similar, but with soil lagging by a month. Average canopy air temperature is 18.6°C, and the coldest month is January (averaging 16.7°C); below minimum temperature (18°C) for rubber-tree growth (Supplementary Figures S1 and S2). The weather data are from a nearby meteorological station. In January and February, affected by both the low temperature and precipitation, soil moisture content and temperatures are low, reaching 21.9% and 18.8°C (Supplementary Figure S2). This period presents stresses in both temperature and drought.

Our study included sites with monoculture rubber plantations with nine different ages in a chronosequence (1–48 years), which were 1, 5, 8, 10, 15, 23, 32, 40, and 48 years. The rubber tree diameters at breast height (1.3 m) at different plantation ages are presented in Supplementary Figure S4 and Supplementary Table S1. At each rubber plantation-age site, a 30 m × 30 m plot was established, containing nine sampling subplots, three on each slope position (upper slope, middle slope, lower slope; Li et al., 2013). All the sites were located on a yellow latosol developed on Permian sandstones at elevations from 560 to 750 m. The vegetation characteristics and management measures (fertilization, weed removal, and latex harvest) of the rubber plantation at different plantation ages are presented in Supplementary Table S1.

### Collection and Analysis of Fresh Mature Leaves and Senescent Leaves

On January 8, 2014, fresh mature leaf samples were collected from five different rubber trees at each plantation-age site using a 10-m-long branch clipper and a retractable ladder (8–16 m high); each sample was mixed with 20 leaves from the bottom one-third of twigs located on opposite sides of the crown of each rubber tree (Rouhi-Moghaddam et al., 2008). Leaf samples were dried at 70°C to a constant weight (at least 48 h) and were then ground in a cleaned agate mortar.

Senescent leaf samples were collected on February 8, 2014 in 0.5 m × 0.5 m litter traps. The litter traps were placed beneath crowns of trees previously sampled at the beginning of leaf-fall season at each site (Rostamabadi et al., 2010). The senescent leaf samples were also dried at 70°C to a constant weight and were then ground in a cleaned agate mortar. The powdered mature and senescent leaf materials were chemically analyzed.

We specifically conducted this research in the early leaf budding period, the amount of leaf buds was very low, so the nutrient content of leaf buds was neglected in this study.

Four nutrient variables were chosen for the analysis of the leaf and litter-fall samples: total carbon (TC), total nitrogen (TN), total phosphorus (TP), and total potassium (TK). The variables were measured according to the protocols given in Supplementary Table S2.

### Soil Sampling and Analysis

Soil sampling was conducted on January 8, 2014 (mature leaf period), February 8, 2014 (end of deciduous period), and March 8, 2014 (leaf budding period). These three sampling time did not match conventional fertilization periods (April and September; Supplementary Table S1), to exclude effects of fertilization on soil nutrients. At the nine subplots of each rubber plantation site, two composite soil samples (approximately 500 g) were obtained from every subplot with a soil auger to a depth of 30 cm after the litter-fall and/or grass layer was carefully removed because the soil layer at 0–30 cm accounts for 80% of the rubber tree vertical root distribution (Srinivasan et al., 2004). After removing all visible stones and plant debris including roots, each fresh soil sample was sieved through 2 mm and maintained at 4°C. All soil microbial related and water soluble nutrient analyses were carried out within 2 weeks on fresh samples (Li et al., 2013).

As total retranslocated nutrients from mature leaves and soil nutrients are very different, plant-nutrient retranslocation may not cause detectable changes in total soil nutrient pools, therefore we used changes in water-soluble soil-nutrient pools to examine nutrient retranslocation effects on soil nutrient properties.

Eight soil variables from three functional subgroups were chosen. The first subgroup included four water-soluble soil-nutrient-related variables: water-soluble nitrate and ammonium nitrogen (WS-NO_3_^-^ and WS-NH_4_^+^; dry soil:deionized water = 1:2.5, Vario MAX CN, Elementar Analysensysteme GmbH, Hanau, Germany), total water-soluble nitrogen (WS-N) is equal to the sum of WS-NO_3_^-^ and WS-NH_4_^+^, water-soluble phosphorus (WS-P), and water-soluble potassium (WS-K; dry soil:deionized water = 1:2.5, HNO_3_-HClO_4_/HCl digestion and inductively coupled plasma-atomic emission spectrometer (ICP-AES) analysis, iCAP6300, Thermo Fisher Scientific, USA; Liu et al., 1996). The second subgroup included three soil microbial structure-related variables: soil microbial biomass carbon (SMB-C), soil microbial biomass nitrogen (SMB-N; fumigation-extraction and total organic carbon analysis, Vario MAX CN, Elementar Analysensysteme GmbH, Hanau, Germany; Vance et al., 1987; Wu et al., 1990, 2006), and the ratio of SMB-N to SMB-C (SMB-N/C). The third subgroup included a soil microbial function-related variable: soil total microbial activity [TMA; fluorescein diacetate (FDA) hydrolysis]. Schnürer and Rosswall (1982) found that the spectrophotometric determination of the hydrolysis of FDA was a simple, sensitive, and rapid method for determining microbial activity in soil. The variables were measured according to the protocols given in the Supplementary Table S2.

### NRE Calculation

Using the data from fresh mature leaves and leaf litter along with the soil nutrient data from the earlier and later deciduous leaf periods, we calculated NRE to account for return of nutrients to rubber plantation soils and indirectly interpreted nutrient use efficiency of the rubber tree along the plantation chronosequence (Eqs. 1–3). The percent NRE was modified based on Finzi et al. (2001) and Huang et al. (2007).

Although changes in foliar nutrient concentrations can reflect both influx and eﬄux of nutrients, they are inadequate as a quantitative measure of nutrient retranslocation unless concomitant changes in leaf mass are also taken into account (Nambiar and Fife, 1991; Saur et al., 2000), thus we used the leaf nutrient stocks instead of nutrient concentrations in this study. In the calculation of all nutrient elements of mature leaves, we used total biomass of senescent leaves to convert the biomass of mature leaves, based on the premise that carbon contents of mature and senescent leaves are relatively stable. Furthermore, we multiplied by a correction factor of 1.25 the actual SNRE calculated on the basis of the 0- to 30-cm soil property data because the 0- to 30-cm soil layer accounted for 80% of rubber tree vertical root distributions (Srinivasan et al., 2004).

To estimate comprehensive retranslocation efficiency (CNRE) of all nutrients (N, P, and K), we calculated CNRE based on the average NRE values of N, P, and K during our study period (1–48 years). The CNRE was defined in Eq. 4 as follows:

$$LNRE(\%)=\frac{A-B}{A}\times 100\%\;\;\;\;\;\;\;\;\;\;\;\;\;\;\;\;\;\;\;\;\;\;\;\;\;\;\;\;\;\;\;\;\;\;\;\;\;\;\;(1)$$

$$SNRE(\%)=\frac{S_{D}-S_{M}}{A}\times 100\%\;\;\;\;\;\;\;\;\;\;\;\;\;\;\;\;\;\;\;\;\;\;\;\;\;\;\;\;\;\;\;\;\;\;\;\;\;\;\;(2)$$

TNRE(%)=LNRE−SNRE                                       (3)

$$C\,N\,R\,E=\frac{1}{n}\times \overset{n}{\underset{i=1}{\Sigma }}\,N\,R\,E_{i}\,\;\;\;\;\;\;\;\;\;\;\;\;\;i=1,2,3\,\;\;\;\;\;\;\;\;\;\;\;\;\;\;\;\;\;\;\;\;\;\;\;\;\;\;\;\;\;\;\;\;\;\;\;\;\;\left(4\right)$$

where LNRE is the total NRE of the mature leaves;

SNRE is the percentage of the LNRE from mature leaves to soil;

TNRE is the percentage of the LNRE from mature leaves to tree;

CNRE is the comprehensive retranslocation efficiency of N, P, and K;

*A* is the nutrient stock of mature leaves;

*B* is the nutrient stock of senescent leaves;

*S*_D_ is the soil nutrient stock after the deciduous-leaf period;

*S*_M_ is the soil nutrient stock during the mature-leaf period; and

NRE*i* is the NRE value of the *i*th nutrient property.

### Data Analysis

For all analyses, every site was evaluated with nine replicates (*N* = 9); sub-samples were collected from every site, and means of sub-samples was used. A Pearson correlation was used to assess correlations among leaf nutrients and soil properties across all sites. One-way analysis of variance (ANOVA) was used to test whether physical, chemical, and biological properties of leaves and soils varied among all sites. The least significant difference test was used to assess pair-wise differences (at 5% levels). All the statistical analyses were conducted using the statistical package SPSS 17.0 (SPSS Inc., Chicago, IL, USA).

## Results

### Nutrient Concentrations during the Mature and Deciduous Leaf Periods

**FIGURE 2:**
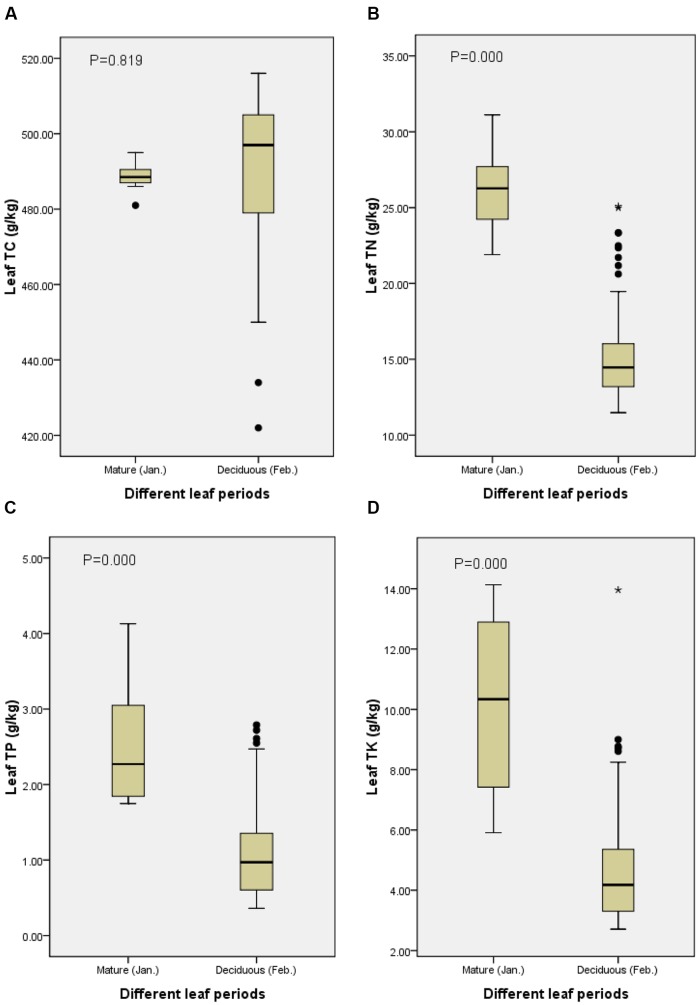
**Comparison of the average concentrations of (A)** total carbon (TC), **(B)** total nitrogen (TN), **(C)** total phosphorus (TP), and **(D)** total potassium (TK) in live and senescent leaves.

Total rubber-tree leaf carbon was 488.50 ± 1.45 mg.kg^-1^ (mean ± SE) during mature leaf period and 490.04 ± 2.32 mg.kg^-1^ during deciduous leaf period. From mature leaf period to deciduous leaf period, no significant changes occurred in rubber-tree leaf TC (*P* = 0.819; **Figure 2**).

Patterns for rubber-tree leaf TN, TP, and TK contents differed from TC. Leaf TN, TP, and TK contents were significantly higher in live mature leaves than in senescent leaves (**Figure 2**). Leaf TN content was 26.18 ± 1.02 mg.kg^-1^ in mature leaf period and 15.51 ± 0.39 mg.kg^-1^ in deciduous leaf period (*P* = 0.000). Leaf TP content was 2.53 ± 0.30 mg.kg^-1^ in mature leaf period and 1.08 ± 0.07 mg.kg^-1^ in deciduous leaf period (*P* = 0.000). Leaf TK content was 10.17 ± 1.12 mg.kg^-1^ in mature leaf period and 4.80 ± 0.24 mg.kg^-1^ in deciduous leaf period (*P* = 0.000; **Figure 2**).

### Characteristics of Water-Soluble Soil-Nutrient Status

**FIGURE 3:**
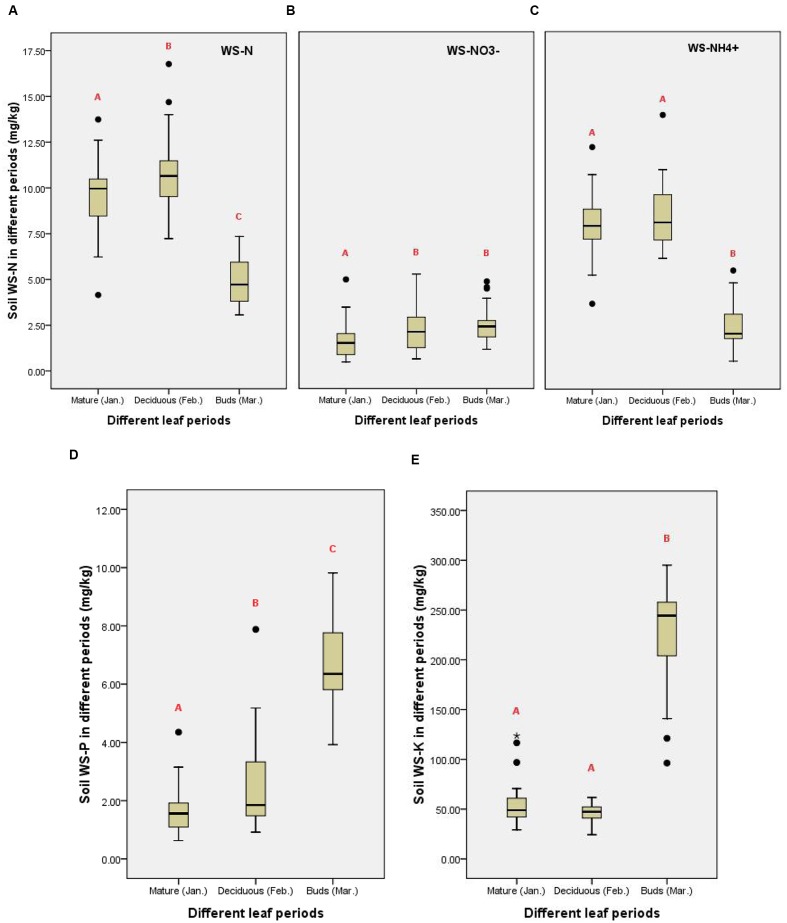
**Average soil total nitrogen (TN) (A–C)**, total phosphorus (TP) **(D)**, and total potassium (TK) **(E)** during different leaf periods (Significant differences between different red alphabet “A, B, and C”).

The WS-N, WS-P, and WS-K in the 0–30 cm soil layer differed significantly among the three leaf periods and exhibited different patterns (**Figure 3**). Concentration of soil WS-N was 9.48 ± 0.41 mg.kg^-1^ during mature leaf period (January), 10.74 ± 0.41 mg.kg^-1^ during deciduous leaf period (February), and 4.89 ± 0.26 mg.kg^-1^ during leaf budding period (March). The value for deciduous leaf period was significantly higher than that for mature leaf period (*P* = 0.018); WS-N content during leaf budding period was significantly lower than that in deciduous leaf period (*P* = 0.000; **Figure 3**, WS-N). From mature leaf period to deciduous leaf period, the difference in soil WS-N was primarily due to changes in soil WS-NO_3_^-^ content (*P* = 0.024; **Figure 3**, WS-NO_3_^-^); however, the difference in soil WS-N between deciduous leaf period and leaf budding period was primarily due to changes in soil WS-NH_4_^+^ content (*P* = 0.000; **Figure 3**, WS-NH_4_^+^).

Soil WS-P was 1.68 ± 0.16 mg.kg^-1^ during mature leaf period, 2.62 ± 0.32 mg.kg^-1^ during deciduous leaf period, and 6.69 ± 0.29 mg.kg^-1^ in leaf budding period. Patterns for soil WS-P content was similar to soil WS-N from mature leaf period and deciduous leaf period, exhibiting a significant increase (*P* = 0.014). Between deciduous leaf period and leaf budding period, there were also significant differences (*P* = 0.000). However, WS-P pattern differed from soil WS-N; its concentration continued to increase during through time and did not decrease as did soil WS-N (**Figure 3**).

Soil WS-K was 55.02 ± 4.48 mg.kg^-1^ during mature leaf period, 45.92 ± 1.84 mg.kg^-1^ during deciduous leaf period, and 226.25 ± 9.48 mg.kg^-1^ during leaf budding period. Patterns of soil WS-K content differed from those of soil WS-N and WS-P contents. Comparing mature leaf period and deciduous leaf period, there were no significant differences (*P* = 0.298); changes occurred only from deciduous leaf period to leaf budding period. Soil WS-K concentrations during leaf budding period were significantly higher than during deciduous leaf period (*P* = 0.000; **Figure 3**).

### Nutrient Retranslocation

Due to presence of positive and negative calculated values, we defined decreases in leaf nutrients and increases in tree body and soil nutrients as a positive direction for the mature and deciduous periods, consistent with the direction of retranslocation processes of rubber trees in the schematic (**Figure 1**).

For nitrogen, the NRE of leaves was 40.77% from mature leaf period to deciduous leaf period (LNRE = 40.77%; **Table 1**; Supplementary Table S3). Among them, N retranslocation from leaves to the soil was 6.50% (SNRE = 6.5%; **Table 1**; Supplementary Table S3). Therefore, retranslocation from leaves to the tree body was 34.27% (TNRE = 40.77% - 6.5% = 34.27%; **Table 1**; Supplementary Table S3). The nitrogen in the leaves was mainly returned to the tree body (34.27%/40.77% = 84.06%).

**Table 1:** Nutrient retranslocation efficiency (NRE) of different nutrient properties in the leaves, soil, and tree body along a rubber plantation chronosequence.

NRE	Nutrient property and location	Plantation age (years)								Average
			
		5	8	10	15	23	32	40	48	
Leaf NRE (%)	Nitrogen (N)	46.11	44.04	52.71	41.78	34.36	27.07	41.06	41.55	40.77
	Phosphorus (P)	68.92	44.59	82.54	70.31	42.47	18.04	58.57	65.83	57.23
	Potassium (K)	44.24	51.74	49.58	20.41	42.05	55.74	67.51	73.82	52.78
Soil NRE (%)	Nitrogen (N)	-5.05	28.10	4.59	13.85	4.80	13.64	2.06	6.95	6.50
	Phosphorus (P)	159.96	191.84	11.95	37.71	18.18	17.61	45.09	69.52	50.45
	Potassium (K)	-113.59	253.31	-311.05	5.26	70.73	-441.24	29.51	-147.53	-121.35
Tree body NRE (%)	Nitrogen (N)	51.17	15.95	48.11	27.93	29.56	13.43	39.00	34.60	34.27
	Phosphorus (P)	-91.04	-147.25	70.59	32.59	24.29	0.43	13.48	-3.70	6.78
	Potassium (K)	157.83	-201.57	360.63	15.14	-28.68	496.98	38.00	221.35	173.13
CNRE (%)	Leaf	53.09	46.79	61.61	44.17	39.63	33.62	55.71	60.40	50.26
	Soil	13.77	157.75	-98.17	18.94	31.24	-136.66	25.55	-23.69	-21.47
	Tree body	39.32	-110.96	159.78	25.22	8.39	170.28	30.16	84.09	71.73

Regarding phosphorus, NRE of leaves was 57.23% from mature leaf period to deciduous leaf period (LNRE = 57.23%; **Table 1**; Supplementary Table S3). During these periods, retranslocation from leaves to soil was 50.45% (SNRE = 50.45%; **Table 1**; Supplementary Table S3). Retranslocation from leaves to the tree body was 6.78% (TNRE = 57.23% - 50.45% = 6.78%; **Table 1**; Supplementary Table S3). Distribution patterns of phosphorus were opposite to those of nitrogen. Phosphorus was mainly transferred to the soil (50.45%/57.23% = 88.15%; **Table 1**; Supplementary Table S3) and not to the rubber-tree body.

For potassium, NRE of leaves was 52.78% from mature leaf period to deciduous leaf period (LNRE = 52.78%; **Table 1**; Supplementary Table S3). However, in contrast to nitrogen and phosphorus patterns, the change in soil potassium was -121.35% (SNRE = -121.35%; **Table 1**; Supplementary Table S3). This negative value indicates that potassium content of soil decreased rather than increased and that loss was greater than TK present in original leaves (121.35% > 100%). This finding indicates that there was no potassium redistribution from leaves into the soil; instead, potassium in soil was absorbed into the tree body in advance, indicating that rubber trees started to absorb and accumulate potassium before leaves began to wither and fall, rather than beginning this stage after defoliation and before sprouting, as we expected. However, with dual supply from leaves and soil, NRE of potassium in the tree body was high, at 173.13% (52.78% + 121.35% = 173.13%; **Table 1**; Supplementary Table S3).

Regarding CNRE, similar to LNRE of N, P, and K ranging from 40.77 to 57.23%, leaf CNRE was 50.26% (**Table 1**; Supplementary Table S3). As a whole, approximately 50.26% of the nutrients were retranslocated from rubber leaves during senescence and withering stage. At the same time, this result also indicates that nearly 50% (100% - 50.26% = 49.74%) of the leaf nutrients remained in senescent leaves and litter. Soil CNRE was -21.47% (**Table 1**; Supplementary Table S3); during the process of leaf nutrient retranslocation, soil nutrients not only did not increase but also decreased by 21.47% overall. This result was mainly due to low retranslocation of nitrogen, and at the same time, potassium did not retranslocate but was absorbed by the tree body. Therefore, CNRE of the rubber tree body was high, at 71.73% (50.26% + 21.47% = 71.73%; **Table 1**; Supplementary Table S3).

### Characteristics of Soil Microbiota-Related Properties

The SMB-C was 108.81 ± 14.81 mg.kg^-1^ during mature leaf period (January), 75.84 ± 11.78 mg.kg^-1^ during deciduous leaf period (February), and 95.15 ± 12.17 mg.kg^-1^ during leaf budding period (March). There were no significant differences between mature leaf and deciduous leaf periods (*P* = 0.077) or between deciduous leaf period and leaf budding period in SMB-C (*P* = 0.296; **Table 2**).

**Table 2:** Soil microbial-related properties during different leaf periods.

Leaf periods	Microbial species structure-related			Microbial function-related
	SMB-C (mg.kg^-1^)	SMB-N (mg.kg^-1^)	SMB-N/C (%)	TMA (μg.kg^-1^.h^-1^)
Mature (January)	108.81 ± 14.81	12.86 ± 0.88a	21.39 ± 3.69	88.81 ± 4.17
Deciduous (February)	75.84 ± 11.78	14.87 ± 1.28a	36.43 ± 4.87	84.11 ± 2.53
Budding (March)	95.15 ± 12.17	9.70 ± 0.91b	25.85 ± 9.11	80.75 ± 3.06

The SMB-N was 12.86 ± 0.88 mg.kg^-1^ during mature leaf period, 14.87 ± 1.28 mg.kg^-1^ during deciduous leaf period, and 9.70 ± 0.91 mg.kg^-1^ during leaf budding period. There were no significant differences between mature leaf period and deciduous leaf period in soil SMB-N (*P* = 0.175); however, this value in leaf budding period was significantly lower than that in deciduous leaf period (*P* = 0.001; **Table 2**).

The ratio of SMB-N to SMB-C (SMB-N/C) was 21.39 ± 3.69% during mature leaf period, 36.43 ± 4.87% during deciduous leaf period, and 25.85 ± 9.11% during leaf budding period. As with dynamics of SMB-C; there were no significant differences in SMB-N/C between mature leaf and deciduous leaf periods (*P* = 0.097) or between deciduous leaf and leaf budding periods (*P* = 0.241; **Table 2**).

The TMA was 88.81 ± 4.17 μg.kg^-1^.h^-1^ during mature leaf period, 84.11 ± 2.53 μg.kg^-1^.h^-1^ during deciduous leaf period, and 80.75 ± 3.06 μg.kg^-1^.h^-1^ during leaf budding period. Similar to dynamics of SMB-C and SMB-N/C, there were no significant differences in SMB-N/C between mature leaf period and deciduous leaf period (*P* = 0.320) or between deciduous leaf and leaf budding periods (*P* = 0.478; **Table 2**).

## Discussion

Nutrient retranslocation from senescing leaves has important implications and advantages; retranslocated nutrients during senescence are directly available for further plant growth, which makes a species less dependent on current nutrient uptake, and waiting for the lengthy processes of litter-fall decomposition and nutrient mineralization is unnecessary (Staaf and Berg, 1982; Berg, 1986). It has been suggested that increased nutrient retranslocation can increase plant fitness, and as such, it is a major nutrient-conservation mechanism (Chapin, 1980; Chabot and Hicks, 1982; Aerts, 1990, 1996), especially in nutrient-poor environments (Berendse and Aerts, 1987; May and Killingbeck, 1992; Grime, 2001; Gloser, 2005; Marty et al., 2009).

Leaf nutrient retranslocation has been studied in relation to biogeochemical cycling, plant fitness and evolution, and ecological influences. Unlike previous experimental work, here we addressed the rubber tree’s internal nutrient retranslocation process and roles of soil under seasonal drought and cold stresses in a somewhat novel environment for this species. We found that rubber trees demonstrate a clear retranslocation process. Furthermore, the retranslocation efficiency was similar to the results of Aerts (1996), Hawkins and Polglase (2000), Saur et al. (2000), and Kumar and Singh (2005), i.e., approximately one-half of N, P, and K contained in mature leaves were retranslocated during senescence (40.77–57.23%, average 50.26%; **Table 1**). High LNRE suggests that nutrient retranslocation is an important nutrient conservation mechanism and could be an integral part of a rubber tree’s strategy to thrive an unfavorable habitat (related to nutrient shortage, seasonal cold and drought stresses in this study). Enhanced nutrient retranslocation would prolong nutrient residence times in the rubber tree body and allow for survival and fitness through periods of resource shortage or cold and drought stresses (Berendse and Aerts, 1987; Escudero et al., 1992).

Patterns of retranslocation for various nutrients were completely different. A total of 40.77% of leaf nitrogen was retranslocated from mature leaf period to leaf deciduous period (**Table 1**). Only 15.94% was redistributed to soil (6.50%/40.77% = 15.94%; **Table 1**). Therefore, nitrogen in leaves was mainly returned to the tree body (34.27%/40.77% = 84.06%). The ratio of leaf nitrogen redistributed to the soil to that returned to the tree body was 1:6.3. The redistribution pattern of phosphorous was completely opposite from nitrogen. Phosphorous was mainly distributed to the soil (50.45%/57.23% = 88.15%; **Table 1**), with only 11.85% to the rubber tree body. The ratio of leaf phosphorus redistributed to the soil to that redistributed to the tree body was 7.44:1. However, in contrast to nitrogen and phosphorus patterns, potassium content in the soil decreased rather than increased, and the loss was greater than total original potassium in the leaves (121.35% > 100%; **Table 1**). Not only there was no leaf potassium redistribution to soil, but potassium from soil was absorbed into the tree body in advance of new leaf construction.

Based on these comprehensive retranslocation efficiencies of N, P, and K during rubber leaf senescence and withering stages, approximately 50.26% of leaf nutrients and 21.47% of the soil nutrients were redistributed to rubber tree body (**Table 1**). This finding suggests that (a) in nutrient retranslocation process, soil not only received nutrients (N and P) from leaves, but also supplied nutrient (K) to trees in the non-growth stage, that is, our hypothesis about the “bank” role of soil was verified, but more than this, soil plays a dual role, as nutrients supplier and free-withdrawing nutrient “bank” in these stages. (b) Rubber trees started to absorb and accumulate nutrients before leaves began to wither and fall, rather than starting after defoliation and before sprouting, as we expected.

Although N, P, and K exhibited relatively obvious changes in the leaves, tree body, and soil through nutrient retranslocation (from mature leaf period to deciduous leaf period) and nutrient uptake (from the deciduous leaf period to the leaf budding period), there were no significant changes in soil microbial structure-related properties or microbial function-related properties, excepted that the SMB-N was significantly lower in the leaf budding period than in the deciduous leaf period (*P* = 0.001; **Table 2**). This finding indicated that under cold and drought stresses, the change in soil nutrient resources did change these aspects of soil microbial community structure and function.

The simplest estimation of nutrient remobilization can be calculated through the “apparent remobilization” method, which relies on the determination of the amount of total nutrient present in the different plant organs at different times of development (Hocking and Pate, 1977). By calculating differences in the nutrient contents of leaves and soil between the mature leaf and deciduous leaf periods, we can infer the nutrient redistribution process of the rubber plantation to a certain extent. However, to understand the NRE and the role of soil in this post-harvest period accurately, use of isotopic tracers is suggested for future studies where possible. Isotopic labeling allows determination of nutrient fluxes from root uptake and by subtraction, the remobilization of unlabeled nutrient between tissues is a more precise method (Maillard et al., 2015).

Rubber tree is an important economic forest crop. We are deeply concerned about contributions of retranslocated nutrients to total nutrient requirement in the following growth season. What is the role of soil in this process, as the nutrient retranslocation is related to managing fertilization in rubber plantations and for latex production. Furthermore, as Aerts (1996) suggested, we should focus on the mechanisms and regulation of the variation in NRE in future studies; for example, the sink strength, phloem transport rate, water availability, and other factors.

## Conclusion

**FIGURE 4:**
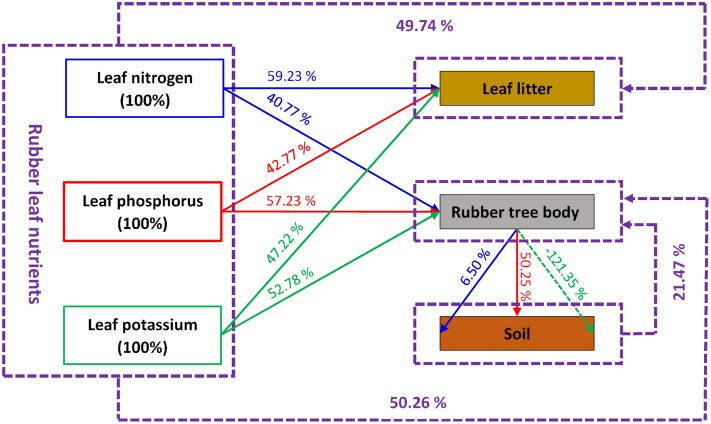
**Retranslocation efficiencies of leaf nitrogen (N), phosphorus (P), potassium (K) and comprehensive retranslocation efficiency of rubber trees from mature leaf period to deciduous leaf period**. The leaf N retranslocation processes and percentages were shown in blue lines and data; the leaf P retranslocation processes and percentages were shown in red lines and data; the leaf K retranslocation processes and percentages were shown in green lines and data; the comprehensive retranslocation processes and percentages were shown in purple lines and data.

Facing annual drought and cold stresses, rubber tree mature leaves have a clear nutrient retranslocation process from leaf senescence period to deciduous period in adaptation to environmental pressures in Xishuangbanna, the northern edge of Asia’s tropics (**Figure 4**). This nutrient retranslocation strategy was an important nutrient conservation mechanism allowing rubber trees to accommodate seasonal cold and drought stresses in this region. In the nutrient retranslocation process, soil plays a dual role as nutrients supplier and free-withdrawing nutrient “bank,” as not only received the nutrients from living mature leaves, but also supplying nutrients to trees during the non-growth stage (**Figure 4**). Nutrient absorption and accumulation began before the leaves started to wither and fall. Soil microorganisms appeared unaffected by nutrient dynamics at this stage.

## Author Contributions

YL, GL, and YX planned and designed the research; YL, GL, and YX performed the experiment and analyzed the data; YL, GL, and YX wrote the manuscript; all authors approved the final manuscript.

## Conflict of Interest Statement

The authors declare that the research was conducted in the absence of any commercial or financial relationships that could be construed as a potential conflict of interest.

## References

[B1] Abdallah M. Dubousset L. Meuriot F. Etienne P. Avice J.-C. Ourry A. (2010). Effect of mineral sulphur availability on nitrogen and sulphur uptake and remobilization during the vegetative growth of *Brassica napus* L. *J. Exp. Bot.* 61 2635–2646. 10.1093/jxb/erq096 20403880 PMC2882259

[B2] Aerts R. (1990). Nutrient use efficiency in evergreen and deciduous species from heathlands. *Oecologia* 84 391–397. 10.1007/bf00329765 28313031

[B3] Aerts R. (1996). Nutrient resorption from senescing leaves of perennials: are there general patterns? *J. Ecol.* 84 597–608. 10.2307/2261481

[B4] Avice J.-C. Etienne P. (2014). Leaf senescence and nitrogen remobilization efficiency in oilseed rape (*Brassica napus* L.). *J. Exp. Bot.* 65 3813–3824. 10.1093/jxb/eru177 24790115

[B5] Berendse F. Aerts R. (1987). Nitrogen-use-efficiency: a biologically meaningful definition? *Funct. Ecol.* 1 293–296.

[B6] Berg B. (1986). Nutrient release from litter and humus in coniferous forest soils - a mini review. *Scand. J. For. Res.* 1 359–369. 10.1080/02827588609382428

[B7] Cao M. Zhang J. H. (1997). Tree species diversity of tropical forest vegetation in Xishuangbanna, SW China. *Biodivers. Conserv.* 6 995–1006. 10.1023/A:1018367630923

[B8] Cao M. Zou X. M. Matthew W. Zhu H. (2006). Tropical forests of Xishuangbanna, China. *Biotropica* 38 306–309. 10.1111/j.1744-7429.2006.00146.x

[B9] Carr M. K. V. (2012). The water relations of rubber (*Hevea brasiliensis*): a review. *Exp. Agric.* 48 176–193. 10.1017/S0014479711000901

[B10] Chabot B. F. Hicks D. J. (1982). The ecology of leaf life spans. *Annu. Rev. Ecol. Syst.* 13 229–259. 10.1146/annurev.es.13.110182.001305

[B11] Chapin F. S. I. I. I. (1980). The mineral nutrition of wild plants. *Ann. Rev. Ecol. Syst.* 11 233–260. 10.1146/annurev.es.11.110180.001313

[B12] Charley J. R. Richards B. N. (1983). “Nutrient allocation in plant communities, mineral cycling in terrestrial ecosystems,” in *Physiological Plant Ecology, IV*, eds Lang O. L. Nobel P. S. Osmund C. B. Zeigler M. (Berlin: Springer), 5–45. 10.1007/978-3-642-68156-1_2

[B13] Chen J.-W. Cao K.-F. (2008). Changes in activities of antioxidative system and monoterpene and photochemical efficiency during seasonal leaf senescence in *Hevea brasiliensis* trees. *Acta Physiol. Plant.* 30 1–9. 10.1007/s11738-007-0070-1

[B14] Chen J.-W. Cao K.-F. (2015). A possible link between hydraulic properties and leaf habits in *Hevea brasiliensis*. *Funct. Plant Biol.* 42 718–726. 10.1071/fp14294 32480715

[B15] De Blécourt M. Brumme R. Xu J. Corre M. D. Veldkamp E. (2013). Soil carbon stocks decrease following conversion of secondary forests to rubber (*Hevea brasiliensis*) plantations. *PLoS ONE* 8:e69357 10.1371/journal.pone.0069357 PMC3716606 23894456

[B16] Dirzo R. Raven P. H. (2003). Global state of biodiversity and loss. *Annu. Rev. Environ. Resour.* 28 137–167. 10.1146/annurev.energy.28.050302.105532

[B17] Escudero A. del Arco J. M. Sanz I. C. Ayala J. (1992). Effects of leaf longevity and retranslocation efficiency on the retention time of nutrients in the leaf biomass of different woody species. *Oecologia* 90 80–87. 10.1007/bf00317812 28312274

[B18] FAO (2010). *Global forest Resources Assessment 2010 FAO Forestry Paper 163*. Rome: FAO.

[B19] Feng Y. Z. (2007). *Man-Made Community.* Kunming: Yunnan Science and Technology Press, 293–295.

[B20] Fife D. N. Nambiar E. K. S. Saur E. (2008). Retranslocation of foliar nutrients in evergreen tree species planted in a Mediterranean environment. *Tree Physiol.* 28 187–196. 10.1093/treephys/28.2.187 18055429

[B21] Finzi A. C. Allen A. S. Delucia E. H. Ellsworth D. S. Schlesinger W. H. (2001). Forest litter production, chemistry, and decomposition following two years of free-air CO2 enrichment. *Ecology* 82 470–484. 10.2307/2679873

[B22] Fischer H. Meyer A. Fischer K. Kuzyakov Y. (2007). Carbohydrate and amino acid composition of dissolved organic matter leached from soil. *Soil Biol. Biochem.* 39 2926–2935. 10.1016/j.soilbio.2007.06.014

[B23] Gardner T. A. Barlow J. Chazdon R. Ewers R. M. Harvey C. A. Peres C. A. (2009). Prospects for tropical forest biodiversity in a human-modified world. *Ecol. Lett.* 12 561–582. 10.1111/j.1461-0248.2009.01294.x 19504750

[B24] Gibson L. Lee T. M. Koh L. P. Brook B. W. Gardner T. A. Barlow J. (2011). Primary forests are irreplaceable for sustaining tropical biodiversity. *Nature* 478 378–381. 10.1038/nature10425 21918513

[B25] Gloser V. (2005). The consequences of lower nitrogen availability in autumn for internal nitrogen reserves and spring growth of *Calamagrostis epigejos*. *Plant Ecol.* 179 119–126. 10.1007/s11258-004-6736-5

[B26] Grime J. P. (2001). *Plant Strategies and Vegetation Processes.* Chichester: John Wiley & Sons Ltd.

[B27] Hawkins B. Polglase P. J. (2000). Foliar concentrations and resorption of nitrogen and phosphorus in 15 species of eucalypts grown under non-limited water and nutrient availability. *Aust. J. Bot.* 48 597–602. 10.1071/BT99036

[B28] Himelblau E. Amasino R. M. (2001). Nutrients mobilized from leaves of *Arabidopsis thaliana* during leaf senescence. *J. Plant Physiol.* 158 1317–1323. 10.1078/0176-1617-00608

[B29] Hocking P. J. Pate J. S. (1977). Mobilization of minerals to developing seeds of legumes. *Annu. Bot.* 41 1259–1278.

[B30] Huang J. Wang X. Yan E. (2007). Leaf nutrient concentration, nutrient resorption and litter decomposition in green broad-leaved forest in eastern China. *For. Ecol. Manag.* 239 150–158. 10.1016/j.foreco.2006.11.019

[B31] Jha K. K. (2014). Temporal patterns of storage and flux of N and P in young Teak plantations of tropical moist deciduous forest, India. *J. For. Res.* 25 75–86. 10.1007/s11676-014-0433-6

[B32] Kumar P. Singh A. (2005). N and P resorption efficiency in certain young tropical tree species planted on mine spoil. *Indian J. For.* 28 371–375.

[B33] Lau C. H. Wong C. B. (1993). “Correction of leaf nutrient values for assessment of Hevea nutrition,” in *Proceedings of the 12th International Plant Nutrition Colloquium: ‘Plant Nutrition-from Genetic Engineering to Field Practice’*, ed. Barrow N. J. (Dordrecht: Kluwer Academic Publishers), 281–283. 10.1007/978-94-011-1880-4_56

[B34] Li H. M. Aide T. M. Ma Y. X. Liu W. J. Cao M. (2007). Demand for rubber is causing the loss of high diversity rain forest in SW China. *Biodivers. Conserv.* 16 1731–1745. 10.1007/s10531-006-9052-7

[B35] Li Y. (2013). *Seasonal Dynamics of Soil Nutrients Under the Tropical Forest Ecosystems in Xishuangbanna, Southwest China*. Ph.D. Dissertation, University of Chinese Academy of Sciences, Beijing.

[B36] Li Y. Deng X. Cao M. Lei Y. Xia Y. (2013). Soil restoration potential with corridor replanting engineering in the monoculture rubber plantations of Southwest China. *Ecol. Eng.* 51 169–177. 10.1016/j.ecoleng.2012.12.081

[B37] Li Y. Xia Y. Lei Y. Deng Y. Chen H. Sha L. (2015). Estimating changes in soil organic carbon storage due to land use changes using a modified calculation method. *iForest Biogeosci. For.* 8 45–52. 10.3832/ifor1151-007

[B38] Li Z. Fox J. M. (2012). Mapping rubber tree growth in mainland Southeast Asia using time-series MODIS 250 m NDVI and statistical data. *Appl. Geogr.* 32 420–432. 10.1016/j.apgeog.2011.06.018

[B39] Limkaisang S. Kom-un S. Furtado E. L. Liew K. W. Salleh B. Sato Y. (2005). Molecular phylogenetic and morphological analyses of *Oidium heveae*, a powdery mildew of rubber tree. *Mycoscience* 46 220–226. 10.1007/s10267-005-0238-8

[B40] Liu G. S. Jiang N. H. Zhang L. D. Liu Z. L. (1996). *Soil Physical and Chemical Analysis and Description of Soil Profiles.* Beijing: Standards Press of China (in Chinese).

[B41] Liu W. Zhu C. Wu J. Chen C. (2016). Are rubber-based agroforestry systems effective in controlling rain splash erosion? *CATENA* 147 16–24. 10.1016/j.catena.2016.06.034

[B42] Liu W. J. Zhang Y. P. Li H. M. Meng F. R. Liu Y. H. Wang C. M. (2005). Fog and rainwater chemistry in the tropical seasonal rain forest of Xishuangbanna, Southwest China. *Water Air Soil Pollut.* 167 295–309. 10.1007/s11270-005-0080-9

[B43] Maillard A. Diquélou S. Billard V. Laîné P. Garnica M. Prudent M. (2015). Leaf mineral nutrient remobilization during leaf senescence and modulation by nutrient deficiency. *Front. Plant Sci.* 6:317 10.3389/fpls.2015.00317 PMC4429656 26029223

[B44] Malagoli P. Laine P. Rossato L. Ourry A. (2005). Dynamics of nitrogen uptake and mobilization in field-grown winter oilseed rape (*Brassica napus*) from stem extension to harvest: I. Global N flows between vegetative and reproductive tissues in relation to leaf fall and their residual N. *Ann. Bot.* 95 853–861. 10.1093/aob/mci091 15701662 PMC4246740

[B45] Marty C. Lamaze T. Pornon A. (2009). Endogenous sink-source interactions and soil nitrogen regulate leaf life-span in an evergreen shrub. *New Phytol.* 183 1114–1123. 10.1111/j.1469-8137.2009.02893.x 19500264

[B46] May J. D. Killingbeck K. T. (1992). Effects of preventing nutrient resorption on plant fitness and foliar nutrient dynamics. *Ecology* 73 1868–1878. 10.2307/1940038

[B47] Millard P. Grelet G.-A. (2010). Nitrogen storage and remobilization by trees: ecophysiological relevance in a changing world. *Tree Physiol.* 30 1083–1095. 10.1093/treephys/tpq042 20551251

[B48] Millard P. Wendler R. Grassi G. Grelet G.-A. Tagliavini M. (2006). Translocation of nitrogen in the xylem of field-grown cherry and poplar trees during remobilization. *Tree Physiol.* 26 527–536. 10.1093/treephys/26.4.527 16414931

[B49] Nambiar E. K. S. Fife D. N. (1987). Growth and nutrient retranslocation in needles of radiata pine in relation to nitrogen supply. *Ann. Bot.* 60 147–156.

[B50] Nambiar E. K. S. Fife D. N. (1991). Nutrient retranslocation in temperate conifers. *Tree Physiol.* 9 185–207. 10.1093/treephys/9.1-2.185 14972864

[B51] Ovington J. D. (1968). “Some factors affecting nutrient distribution within ecosystems,” in *Proceedings of the Copenhagen Symposium: Functioning of Terrestrial Ecosystems of Primary Production Level*, ed. Eckardt F. E. (Paris: UNESCO), 95–105.

[B52] Parzych A. Astel A. Trojanowski J. (2008). Fluxes of biogenic substances in precipitation and throughfall in woodland ecosystems of the Slowinski National Park. *Arch. Environ. Prot.* 34 13–24.

[B53] Powers J. S. Corre M. D. Twine T. E. Veldkamp E. (2011). Geographic bias of field observations of soil carbon stocks with tropical land-use changes precludes spatial extrapolation. *Proc. Natl. Acad. Sci. U.S.A.* 108 6318–6322. 10.1073/pnas.1016774108 21444813 PMC3076837

[B54] Rostamabadi A. Tabari M. Salehi A. Sayad E. Salehi A. (2010). Comparison of nutrition, nutrient return and nutrient retranslocation between stands of *Alnus subcordata* and *Taxodium distichum* in Tashbandan, Amol (Mazandaran). *J. Wood For. Sci. Technol.* 17 65–78.

[B55] Rouhi-Moghaddam E. Hosseini S. M. Ebrahimi E. Tabari M. Rahmani A. (2008). Comparison of growth, nutrition and soil properties of pure stands of *Quercus castaneifolia* and mixed with *Zelkova carpinifolia* in the Hyrcanian forests of Iran. *For. Ecol. Manag.* 255 1149–1160. 10.1016/j.foreco.2007.10.048

[B56] Ryan D. F. Bormann F. H. (1982). Nutrient resorption in northern hardwood forests. *BioScience* 32 29–32. 10.2307/1308751

[B57] Salehi A. Ghorbanzadeh N. Salehi M. (2013). Soil nutrient status, nutrient return and retranslocation in poplar species and clones in northern Iran. *iForest Biogeosci. For.* 6 336–341. 10.3832/ifor0976-006

[B58] Saur E. Nambiar E. K. S. Fife D. N. (2000). Foliar nutrient retranslocation in *Eucalyptus globulus*. *Tree Physiol.* 20 1105–1112. 10.1093/treephys/20.16.1105 11269962

[B59] Schnürer J. Rosswall T. (1982). Fluorescein diacetate hydrolysis as a measure of total microbial activity in soil and litter. *Appl. Environ. Microbiol.* 43 1256–1261. 16346026 10.1128/aem.43.6.1256-1261.1982 PMC244223

[B60] Singh L. Singh J. S. (1991). Storage and flux of nutrients in dry tropical forest in India. *Ann. Bot.* 68 275–284.

[B61] Sodhi N. S. Koh L. P. Brook B. W. Ng P. K. L. (2004). Southeast Asian biodiversity: the impending disaster. *Trends Ecol. Evol.* 19 654–660. 10.1016/j.tree.2004.09.006 16701328

[B62] Srinivasan K. Kunhamu T. K. Mohan Kumar B. (2004). Root excavation studies in a mature rubber (*Hevea brasiliensis* Muell. Arg.) plantation. *Nat. Rubber Res.* 17 18–22.

[B63] Staaf H. Berg B. (1982). Accumulation and release of plant nutrients in decomposing Scots pine needle litter. Long-term decomposition in a Scots pine forest II. *Can. J. Bot.* 60 1561–1568. 10.1139/b82-199

[B64] Vance E. D. Brookes P. C. Jenkinson D. S. (1987). An extraction method for measuring soil microbial biomass C. *Soil Biol. Biochem.* 19 703–707. 10.1016/0038-0717(87)90052-6

[B65] Wu J. Joergensen R. G. Pommerening B. Chaussod R. Brookes P. C. (1990). Measurement of soil microbial biomass, by fumigation–extraction - an automated procedure. *Soil Biol. Biochem.* 22 1167–1169. 10.1016/0038-0717(90)90046-3

[B66] Wu J. Lin Q. M. Huang Q. Y. Xiao H. A. (2006). *Determinations and Applications of Soil Microbial Biomass.* Beijing: China Meteorological Press.

[B67] Wu J. Liu W. Chen C. (2016). Can intercropping with the world’s three major beverage plants help improve the water use of rubber trees? *J. Appl. Ecol.* 53 1787–1799. 10.1111/1365-2664.12730

[B68] Xu J. Grumbine R. E. Beckschäfer P. (2014). Landscape transformation through the use of ecological and socioeconomic indicators in Xishuangbanna, Southwest China, Mekong Region. *Ecol. Indic.* 36 749–756. 10.1016/j.ecolind.2012.08.023

[B69] Zhang J. L. Zhang S. B. Chen Y. J. Zhang Y. P. Poorter L. (2015). Nutrient resorption is associated with leaf vein density and growth performance of dipterocarp tree species. *J. Ecol.* 103 541–549. 10.1111/1365-2745.12392

[B70] Zhou W.-J. Ji H.-L. Zhu J. Zhang Y.-P. Sha L.-Q. Liu Y.-T. (2016). The effects of nitrogen fertilization on N2O emissions from a rubber plantation. *Sci. Rep.* 6:28230 10.1038/srep28230 PMC4915005 27324813

