# Supplementary material for: Rubber Trees Demonstrate a Clear Retranslocation Under Seasonal Drought and Cold Stresses
Source: Front Plant Sci. 2016 Dec 20;7:1907. doi: 10.3389/fpls.2016.01907 (PMC5168426; doi:10.3389/fpls.2016.01907)
Supplement: Supplementary file 1 [file Data_Sheet_1.doc]

**Supporting Information for**

**Rubber trees demonstrate a clear retranslocation under seasonal drought and cold stresses**

Yuwu Li ^a, b, ǂ^, *, Guoyu Lan ^b, ǂ^, *, Yujie Xia ^c, ǂ^, *

^a^ *Key Laboratory of Tropical Forest Ecology, Xishuangbanna Tropical Botanical Garden, Chinese Academy of Sciences, Yunnan, China*

^b^ *Danzhou Investigation & Experiment Station of Tropical Crops, Ministry of Agriculture, Rubber Research Institute, Chinese Academy of Tropical Agricultural Sciences, Hainan, China*

^c^ *Kunming Institute of Zoology, Chinese Academy of Sciences, Yunnan, China*

^ǂ^ *Joint first authors. These authors contributed equally to this work*

*** Corresponding authors:**

Dr. Yuwu Li ([lyw@xtbg.org.cn](mailto:lyw@xtbg.org.cn));

Dr. Guoyu Lan ([langyrri@163.com](mailto:langyrri@163.com));

Mrs. Yujie Xia ([xiayujie@mail.kiz.ac.cn](mailto:xiayujie@mail.kiz.ac.cn)).

Tel.: +86-871-681-25324; Fax: +86-871-681-25321.

*
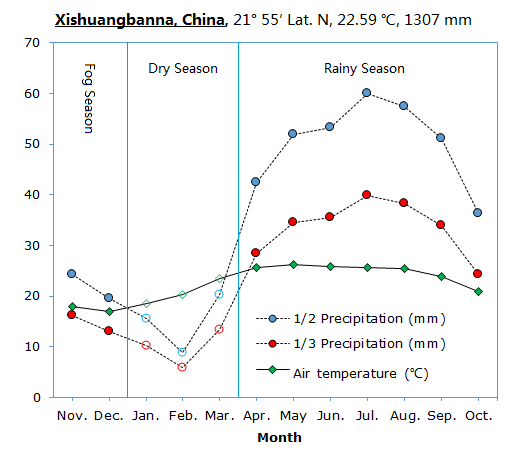
*

Figure S1. Bagnouls-Gaussen ombrothermic diagram of Xishuangbanna (2005-2015).

When monthly precipitation P>100 mm, P is denoted as P’ in the diagram, and P’=100 + (P-100)/10.


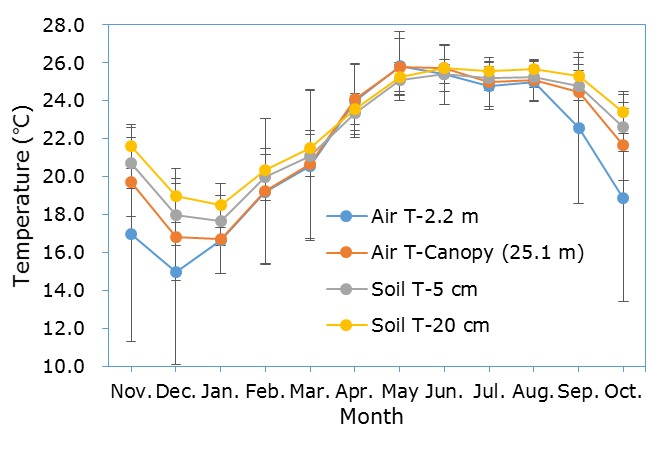

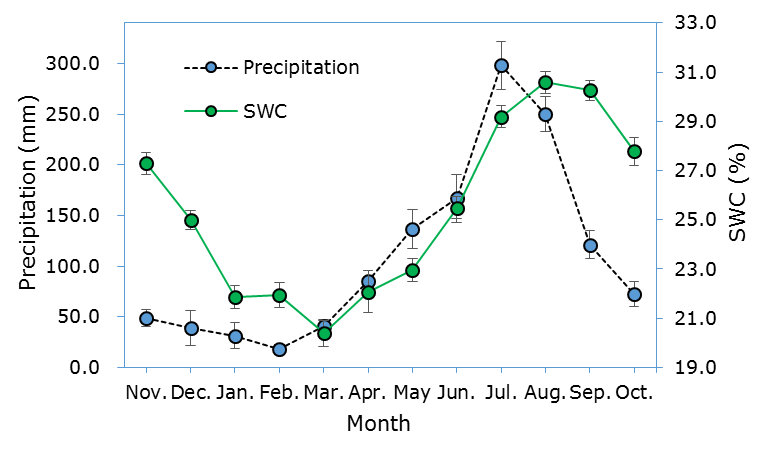


Figure S2. Patterns of air and soil temperatures, precipitation and soil water content of rubber plantation in Xishuangbanna (2010-2015).


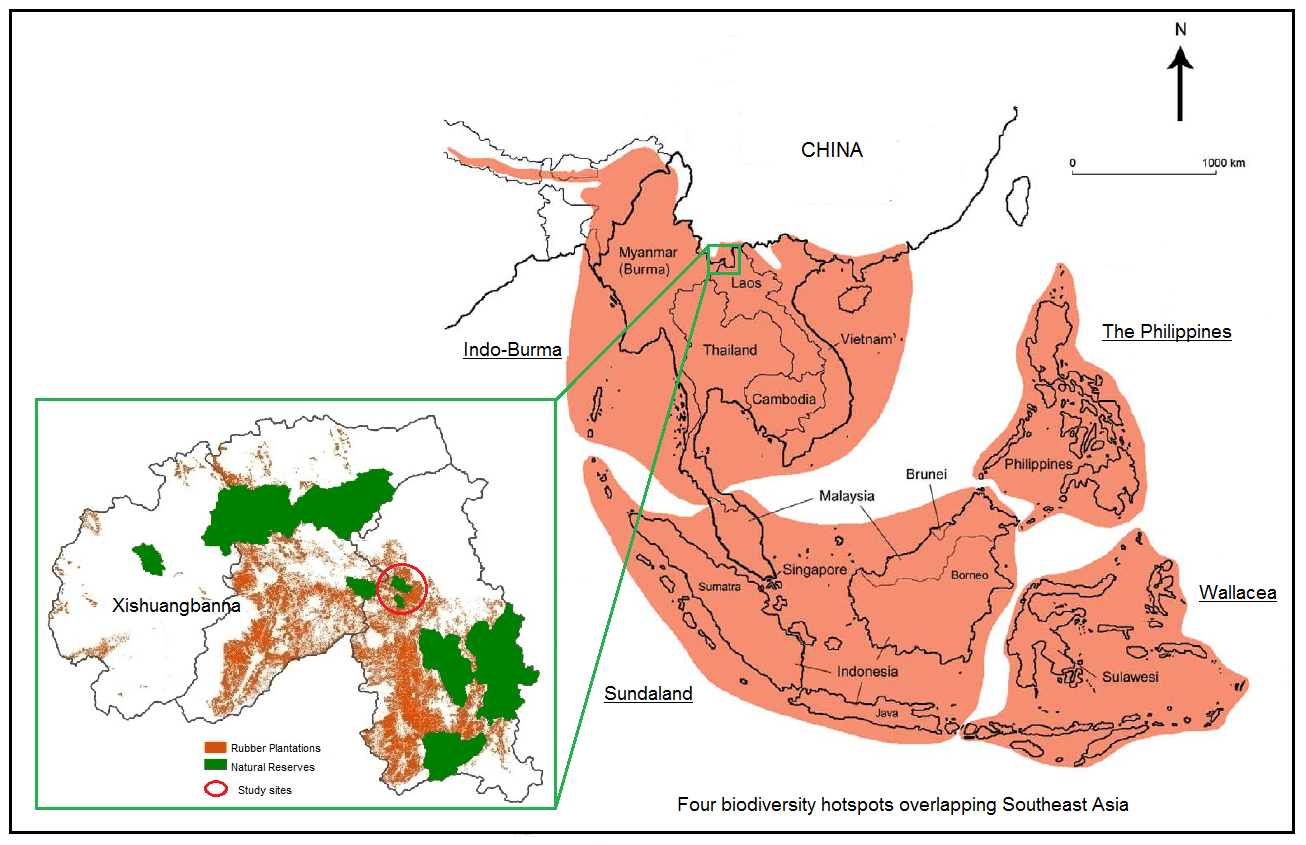


Figure S3. Map of the study sites in Xishuangbanna, the north edge of SE Asian tropics (Sodhi et al. 2004; Li et al. 2013; Xu et al. 2014).


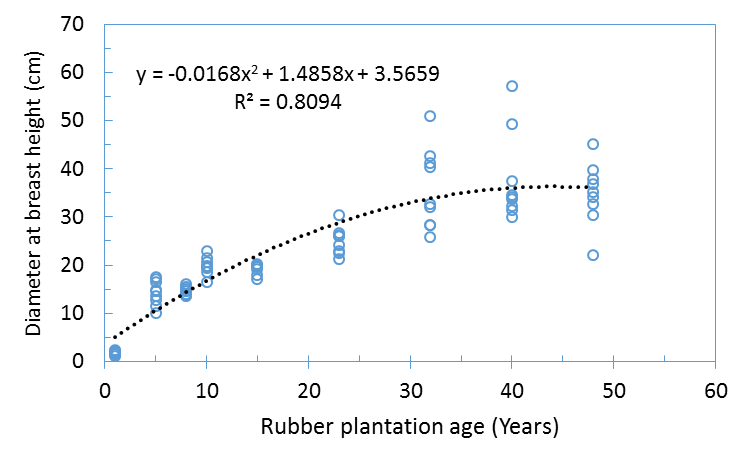


Figure S4. Diameters at breast height of the rubber trees in different ages in Xishuangbanna.

Table S1. Vegetation characteristics and management measures in the rubber plantations along the chronosequence in Xishuangbanna.

| **Vegetation characteristics** | **Rubber plantation age (*A*)** | | | |
| --- | --- | --- | --- | --- |
|  | *A* < 8 years | 8 ≤ *A* < 15 years | 15 ≤ *A* ≤ 30 years | >30 years |
| Production phase | Pre-production period (NP) | Initial production period (IP) | High production period (HP) | Declining production period (DP) |
| Canopy height (m) | <5 m | <12 m | <23 m | <30 m |
| Diameter at breast height (1.3m) (cm) | <15 cm | <20 cm | <36 cm | <39 cm |
| Annually litter-fall (ton ha^-1^) | 3.14 | 4.97 | 3.76 | 4.61 |
| Annually fertilization added (mixed fertilizer (N 20%, P 10%, K 10%)) | None | 2 times (April and September);  180 kg ha^−1^ | 2 times (April and September);  180 kg ha^−1^ | 2 times (April and September);  180 kg ha^−1^ |
| Annual weeding frequency | 1–2 times | 3–4 times | 3–4 times | 3–4 times |
| Annual harvest  (dry rubber latex) | None | 1600 kg ha^−1^ | 2660 kg ha^−1^ | 2000 kg ha^−1^ |

Table S2. Analysis methods for soil variables of the study samples.

| **Variables** | **Method** | **Reference** |
| --- | --- | --- |
| **Leaf nutrient-related** |  |  |
| TC | Vario MAX CN analysis |  |
| TN | Vario MAX CN analysis |  |
| TP | HNO_3_-HClO_4_/HCl digestion and ICP-AES analysis |  |
| TK | HNO_3_-HClO_4_/HCl digestion and ICP-AES analysis |  |
| **Soil nutrient-related** |  |  |
| WS-NO_3_^-^ | Vario MAX CN analysis (dry soil : deionized water, 1:2.5) | Liu et al. 1996 |
| WS-NH_4_^+^ | Vario MAX CN analysis (dry soil : deionized water, 1:2.5) | Liu et al. 1996 |
| WS-P | HNO_3_-HClO_4_/HCl digestion and ICP-AES analysis (dry soil : deionized water, 1:2.5) | Liu et al. 1996 |
| WS-K | HNO_3_-HClO_4_/HCl digestion and ICP-AES analysis(dry soil : deionized water, 1:2.5) | Liu et al. 1996 |
| **Soil microbial** **species structure -related** |  |  |
| SMB-C | Fumigation–extraction and TOC analysis | Vance et al. 1987; Wu et al. 1990; 2006 |
| SMB-N | Fumigation–extraction and TOC analysis | Vance et al. 1987; Wu et al. 1990; 2006 |
| **Soil microbial** **function-related** |  |  |
| TMA | Fluorescein diacetate hydrolysis | Schnürer and Rosswall 1982 |

Table S3. Data of rubber tree leaf and soil nutrient properties during different leaf periods along a plantation chronosequence.

| **Sample name and *NRE*** | **Nutrient property** |  |  | **Rubber plantation age (years)** | | | | | |  | **Average** |
| --- | --- | --- | --- | --- | --- | --- | --- | --- | --- | --- | --- |
|  |  | 1 | 5 | 8 | 10 | 15 | 23 | 32 | 40 | 48 |  |
| Mature leaf  (kg ha^-1^) | Nitrogen  (N) | ND | 45.91±17.52 | 29.47±14.33 | 112.36±13.01 | 72.54±20.52 | 108.49±16.27 | 114.13±20.97 | 79.46±10.48 | 91.52±8.52 | 80.79±15.30 |
|  | Phosphorus  (P) | ND | 5.02±1.91 | 5.27±2.56 | 10.54±1.22 | 5.86±1.66 | 7.04±1.06 | 7.04±1.29 | 6.01±0.79 | 11.09±1.03 | 7.81±1.48 |
|  | Potassium  (K) | ND | 23.62±9.02 | 17.84±8.67 | 39.29±4.55 | 20.14±5.70 | 23.78±3.57 | 32.13±5.90 | 34.16±4.51 | 42.25±3.93 | 31.34±5.95 |
| Senescent leaf  (kg ha^-1^) | Nitrogen  (N) | ND | 24.74±9.44 | 16.49±8.02 | 53.14±6.15 | 42.23±11.94 | 71.21±10.68 | 83.23±15.29 | 46.83±6.18 | 53.49±4.98 | 47.85±9.06 |
|  | Phosphorus  (P) | ND | 1.56±0.60 | 2.92±1.42 | 1.84±0.21 | 1.74±0.49 | 4.05±0.61 | 5.77±1.06 | 2.49±0.33 | 3.79±0.35 | 3.34±0.63 |
|  | Potassium  (K) | ND | 13.17±5.03 | 8.61±4.19 | 19.81±2.29 | 16.03±4.53 | 13.78±2.07 | 14.22±2.61 | 11.10±1.46 | 11.06±1.03 | 14.80±2.80 |
| Soil in mature leaf period (kg ha^-1^) | Nitrogen  (N) | 42.99±10.33 | 45.09±5.53 | 24.59±6.33 | 38.36±4.18 | 39.86±1.78 | 34.90±8.73 | 42.03±2.40 | 50.22±7.00 | 38.30±8.47 | 39.59±8.85 |
|  | Phosphorus  (P) | 5.18±1.40 | 7.92±2.19 | 12.64±5.12 | 4.52±0.06 | 5.14±0.99 | 4.62±2.92 | 5.07±0.66 | 7.59±0.61 | 10.46±2.71 | 7.02±3.41 |
|  | Potassium  (K) | 213.04±48.96 | 275.03±183.91 | 168.99±43.05 | 321.42±73.86 | 222.38±28.52 | 170.39±38.95 | 310.34±184.93 | 203.10±6.05 | 183.55±44.02 | 229.80±97.29 |
| Soil in senescent leaf period (kg ha^-1^) | Nitrogen  (N) | 40.25±8.34 | 42.77±4.31 | 32.87±2.88 | 43.52±0.90 | 49.91±2.96 | 40.11±3.97 | 57.60±4.27 | 51.86±15.94 | 44.66±2.35 | 44.84±8.97 |
|  | Phosphorus  (P) | 6.11±0.84 | 15.95±3.62 | 22.75±9.16 | 5.78±1.59 | 7.35±0.96 | 5.90±1.95 | 6.31±1.12 | 10.30±1.84 | 18.17±4.79 | 10.96±6.92 |
|  | Potassium  (K) | 150.72±16.87 | 248.23±12.16 | 214.18±6.81 | 199.21±11.61 | 223.44±19.39 | 187.21±8.17 | 168.57±23.73 | 213.18±13.8 | 121.22±17.47 | 191.77±39.96 |
| Soil in budding period (kg ha^-1^) | Nitrogen  (N) | 23.85±5.82 | 14.37±1.89 | 16.86±2.49 | 15.83±1.75 | 17.44±6.57 | 18.73±3.97 | 26.03±1.22 | 25.78±4.26 | 24.74±4.77 | 20.40±5.60 |
|  | Phosphorus  (P) | 23.06±3.96 | 30.98±5.05 | 36.81±6.37 | 33.05±5.53 | 22.73±6.08 | 24.84±4.61 | 23.07±2.95 | 28.36±2.59 | 28.50±5.42 | 27.93±6.28 |
|  | Potassium  (K) | 526.54±135.87 | 950.00±141.68 | 972.29±165.61 | 1049.40±27.85 | 1020.18±60.57 | 773.99±175.41 | 1160.77±106.17 | 1061.01±84.99 | 990.40±74.32 | 944.95±205.84 |

**References**

Li Y, Deng X, Cao M, Lei Y, Xia Y (2013) Soil restoration potential with corridor replanting engineering in the monoculture rubber plantations of Southwest China. *Ecological Engineering* **51**, 169-177. doi: 10.1016/j.ecoleng.2012.12.081.

Liu GS, Jiang NH, Zhang LD, Liu ZL (1996) *Soil Physical and Chemical Analysis and Description of Soil Profiles*. Standards Press of China, Beijing, China (in Chinese).

Schnürer J, Rosswall T (1982) Fluorescein diacetate hydrolysis as a measure of total microbial activity in soil and litter. *Applied and Environmental Microbiology* **43**, 1256-1261.

Sodhi NS, Koh LP, Brook BW, Ng PKL (2004) Southeast Asian biodiversity: the impending disaster. *Trends in Ecology and Evolution* **19**, 654-660. doi: 10.1016/j.tree.2004.09.006.

Vance ED, Brookes PC, Jenkinson DS (1987) An extraction method for measuring soil microbial biomass C. *Soil Biology & Biochemistry* **19**, 703-707. doi: 10.1016/0038-0717(87)90052-6.

Wu J, Joergensen RG, Pommerening B, Chaussod R, Brookes PC (1990) Measurement of soil microbial biomass, by fumigation--extraction - an automated procedure. *Soil Biology & Biochemistry* **22**, 1167-1169. doi: 10.1016/0038-0717(90)90046-3.

Wu J, Lin QM, Huang QY, Xiao HA (2006) *Determinations and applications of soil microbial biomass*. China Meteorological Press, Beijing, China (in Chinese).

Xu J, Grumbine RE, Beckschäfer P (2014) Landscape transformation through the use of ecological and socioeconomic indicators in Xishuangbanna, Southwest China, Mekong Region. *Ecological Indicators* **36**, 749-756. doi: 10.1016/j.ecolind.2012.08.023.
